# Problematic Internet Use Was Associated With Psychological Problems Among University Students During COVID-19 Outbreak in China

**DOI:** 10.3389/fpubh.2021.675380

**Published:** 2021-06-15

**Authors:** Xinyan Xie, Kaiheng Zhu, Qi Xue, Yu Zhou, Qi Liu, Hao Wu, Zihao Wan, Jiajia Zhang, Heng Meng, Bing Zhu, Ranran Song

**Affiliations:** ^1^Department of Maternal and Child Health and MOE (Ministry of Education) Key Lab of Environment and Health, School of Public Health, Tongji Medical College, Huazhong University of Science and Technology, Wuhan, China; ^2^Department of Epidemiology and Biostatistics, Arnold School of Public Health, University of South Carolina, Columbia, SC, United States; ^3^Hangzhou Center for Disease Control and Prevention, Hangzhou, China

**Keywords:** coronavirus disease 2019, university students, problematic internet use, posttraumatic stress disease symptoms, depressive symptom, anxiety symptom

## Abstract

**Background:** As the coronavirus disease 2019 (COVID-19) pandemic progressed globally, school closures and home quarantine may cause an increase in problematic Internet use among students in universities. Such a traumatic stress event may also contribute to the development of posttraumatic stress disorder (PTSD), depressive, and anxiety symptoms. This study aimed to evaluate the prevalence of PTSD, depressive, and anxiety symptoms as well as the predictive role of problematic Internet use in the above-mentioned psychological problems in university students.

**Methods:** A cross-sectional study was conducted through an online survey of 8,879 students in China between April 20 and April 26, 2020. The presence of PTSD, depressive, and anxiety symptoms and problematic Internet use were evaluated using PTSD Checklist for DSM-5, the Center for Epidemiologic Studies Depression 9-item scale, the generalized anxiety disorder 7-item scale, and the Young diagnostic questionnaire, respectively. Sociodemographic information and the knowledge, attitude, and practice (KAP) toward COVID-19 data were also collected.

**Results:** A total of 4,834 (54.4%) participants were female, and 7,564 (85.2%) were undergraduate students. A total of 615 students (6.9%) reported PTSD symptoms; 5.2% (465) and 10.1% (896) reported moderate to severe depressive and anxiety symptoms, respectively. The problematic Internet use was significantly associated with higher risk of PTSD, depressive, and anxiety symptoms (odds ratio 2.662 [95% CI, 2.239–3.165], odds ratio 4.582 [95% CI, 3.753–5.611], odds ratio 3.251 [95% CI, 2.814–3.757], respectively; all *P* < 0.001). Lower attitude and practice scores also contributed to the risk of depressive, anxiety, and PTSD symptoms (*P* < 0.05).

**Conclusions:** Psychological problems should be paid more attention, and problematic Internet use may be a predictor when screening high-risk students for psychological problems. Our results will aid in timely psychological screening, which is meaningful in the prevention and intervention of psychological problems.

## Introduction

Since the coronavirus disease 2019 (COVID-19) epidemic progressed in Wuhan, Hubei Province, China, in December 2019, COVID-19 has become a global pandemic and has been declared a Public Health Emergency of International Concern by the World Health Organization (1). According to monitoring data of the United Nations Educational, Scientific and Cultural Organization, this infection has caused 195 country-wide closures and affected 157,833,678,8 learners, accounting for 90.1% of the total enrolled learners (2). In China, 280 million students were restricted to their homes and had online class during the COVID-19 outbreak (3).

COVID-19 itself, and the school closures and home quarantine caused by COVID-19, are traumatic stress events for most students, especially those with preexisting emotional disorders and those from vulnerable families (4). Such experiences could contribute to the development of posttraumatic stress disorder (PTSD) symptoms, depression, and psychological distress, which was observed during the epidemic of severe acute respiratory syndrome (SARS) (5) and the novel H1N1 influenza (6). The Centers for Disease Control and Prevention (CDC) claimed that mental health is part of the mission of addressing communicable disease (7). As for COVID-19, the prevalence of PTSD, depressive, and anxiety symptoms ranged from 7 to 27.39%, 3.7 to 48.14%, and 3.8 to 38.48%, respectively, and the participants included the general population (8–10), medical staff (11), the workforce (12), the affected population (13), adolescents (14), and youth (15). Research found that a majority of participants (71.26%) indicated that their stress/anxiety levels had increased during the pandemic (16). In addition, 4.18 and 3.41% of Chinese university students reported moderate to severe depressive and anxiety symptoms, respectively (17). Tang et al. (18) reported that the prevalence of PTSD symptoms in 2,485 Chinese university students about 1 month after the COVID-19 outbreak was 2.7%. However, PTSD may have a delayed onset, which may be missed in the initial stage (19). A 30-day prevalence is valuable, though a sufficiently large sample size is needed for estimation (20). Factors such as previous diagnoses of mental health problems, bad sleep quality, and younger age group contributed to the higher risk for psychological problems (8, 9, 17).

In addition to the above risk factors found in recent studies, individuals may be more susceptible to mental health problems if they suffer from addictive problems, including problematic Internet use. According to a theory proposed by Young et al., problematic Internet use might occur to compensate for the negative effects of life, even though it is still a problematic behavioral pattern (21). Individuals with problematic Internet use were 2.77 times and 2.70 times more likely to suffer from depression and anxiety, respectively, than those without problematic Internet use. The prevalence of psychiatric co-morbidity in problematic Internet use is similar to that in substance use and addictive disorders (22). Problematic Internet use may be associated with increased social isolation, which could lead to depression (23). Studies also observed that the psychological components of anxiety such as sensitivity to stress were relative to the addictions (24).

When exposed to traumatic experiences, one may use addictive behaviors to avoid reminders of the trauma and cope with the PTSD symptoms (25). The emotion regulation partially explained the association between PTSD and addictive behaviors (26). Furthermore, the avoiding or negative coping styles, such as addictive behaviors, when facing emergencies, are related to mental health problems (27). For instance, 7.3% of a population could be expected to report increased alcohol consumption in the aftermath of the September 11, 2001, terrorist attacks in New York City (28), and this could be the predictor of psychological problems like depression, anxiety, and PTSD (29). Considering the similar basic mechanisms between behavioral addiction (i.e., problematic Internet use) and substance abuse, problematic Internet use may be a maladaptive method of coping with PTSD symptoms, which was similar to the proposed association between PTSD and substance-use disorders. Studies also have found that problematic Internet use was significantly and independently associated with a high level of PTSD symptoms in students following the Sewol ferry disaster in South Korea (30).

Meanwhile, during the COVID-19 outbreak, university students stayed at home and had a lot of time to spend online. The World Health Organization reported that excessive e-device use was arguably a type of behavioral addiction that presented as a repetitive pattern of behavioral engagement in a specific area (31). Young adults are in a period of major changes in social roles and responsibilities, such as transition from compulsory education to university and establishing a romantic relationship (32). Mental health problems were highly prevalent among young adults before the COVID-19 outbreak. The Report on National Mental Health Development in China (2019–2020) showed that 18.5 and 8.4% of university students had depressive and anxiety symptoms, respectively (33). Nearly 47% of the university students suffered from different kinds of psychological problems, according to a national survey on the psychological well-being of Chinese university students in 2019 conducted by Dingxiangyuan and China Youth Daily (34). The meta-analysis study showed that the prevalence of depression among Chinese students ranged from 3 to 80.6% (35). The percentage of U.S. college students with a diagnosed mental health condition increased from 21.9% in 2007 to 35.5% in 2016–2017 (36). Studies have also indicated that excessive Internet use is related to psychological health (37, 38). Social media exposure is positively associated with high odds of anxiety and depression in the general population during the COVID-19 period (39, 40). Excessive Internet use among Chinese children and adolescents was observed during the outbreak of COVID-19 (41). And COVID-19 anxiety was found to be correlated with the severity of problematic Internet use (42). It was unclear to what extent the psychological problems worsened and what impact prolonged Internet use had on mental health during the COVID-19 pandemic. We hypothesized that the mental health status deteriorated among university students during the COVID-19 period and that problematic Internet use was associated with the psychological problems. We conducted a cross-sectional study to explore the prevalence of PTSD, depressive, and anxiety symptoms in 8,879 students from 23 universities in China ~3 months after the COVID-19 outbreak in China, as many individuals develop symptoms within 3 months of the trauma (43). The relationship between problematic Internet use and PTSD, depressive, and anxiety symptoms was evaluated.

## Materials and Methods

### Participants

A snowball sampling method was used to invite undergraduate and graduate students to participate in the online survey between April 20 and April 26, 2020, ~3 months after the COVID-19 outbreak in China. A total of 9,044 participants completed the survey through an online crowdsourcing platform called Wenjuanxing in mainland China, which provides functions equivalent to Amazon Mechanical Turk. The survey link was sent to the students' cellphones and the statement “I agree to participate in the survey voluntarily” was presented to the participant before the survey. The students proceeded to the survey after they had consented.

### Measures

The demographic characteristics of participants were collected including age, sex, the location during the COVID-19 outbreak, residential district (urban and rural), type of students (undergraduate students, master's degree candidates, and doctoral candidates), whether there was anyone around them infected with COVID-19, and the knowledge, attitude, and practice (KAP) toward COVID-19. The detailed questions about KAP were showed in Supplementary Table 1. The Young diagnostic questionnaire (YDQ) consisting of eight items was used to assess the Internet use (i.e., “Do you stay online longer than originally intended?”). Participants who answer “yes” to five or more items are categorized as having problematic Internet use. The translated version, revised by Lv Ye, was directly used in the study, as it was widely used in China (44). The Cronbach's coefficient of YDQ was 0.805 in the study. After the exploratory factor analysis, the Kaiser-Meyer-Olkin (KMO) coefficient was 0.874 and Bartlett test *P*-value was < 0.001.

The PTSD Checklist for DSM-5 (PCL-5) is a 20-item self-report questionnaire (45). The participants are asked to fill out the checklist in relation to a specific event in the past month, that was, the COVID-19 epidemic (i.e., “In the past month, how much were you been bothered by: ‘Repeated, disturbing, and unwanted memories of the stressful experience?”'). The rating scale is 0–4 for each symptom corresponding to “Not at all,” “A little bit,” “Moderately,” “Quite a bit,” and “Extremely.” We used the Chinese version of PCL-5, which has been adapted by translation and back translation (46). Probable PTSD diagnoses are made using the DSM-5 criteria that requires at least one item from cluster B (reexperiencing), one item from cluster C (avoidance), two items from cluster D (negative thoughts and feelings) and two items from cluster E (Hyperarousal). The items with a score of two or higher were considered clinically relevant (45). The Cronbach's coefficient of PCL-5 was 0.968 in the study. The KMO coefficient was 0.976 and Bartlett's Test of Sphericity was < 0.001.

The Chinese version of the Center for Epidemiologic Studies Depression 9-item scale (CES-D9) was used to measure the level of depressive symptomatology which was developed by He et al. and was proven to be reliable and valid (47). Respondents were asked to rate each item to indicate how often they had felt that way over the previous week on a 4-point scale: 0 (rarely; <1 day), 1 (some of the time; 1–2 days), 2 (a moderate amount of the time; 3–4 days), or 3 (most or all of the time; 5–7 days) (i.e., “I have a good life”). A higher total score indicates a higher severity of depressive symptoms. A cut-off value of 17 was used (47). In this study, Cronbach's coefficient and KMO coefficient of the CES-D9 were 0.883 and 0.869, respectively. Bartlett test *P*-value was < 0.001.

The generalized anxiety disorder 7-item scale (GAD-7) published by Spitzer (48), was used to measure anxiety symptoms. Participants are asked how often, during the last 2 weeks, they have been bothered by the symptoms of items (i.e., “Feeling nervous, anxious, or on edge”). Response options are “not at all,” “several days,” “more than half the days,” and “nearly every day,” scored as 0, 1, 2, and 3, respectively. The Chinese version of GAD-7 has proven to be reliable and valid (49). A higher total score indicates a higher severity of anxiety symptoms. A cut-off point used in this study was 10 (48). GAD-7 showed good internal consistency and in the study with a Cronbach's coefficient of 0.936. The KMO value was 0.926 and Bartlett's Test of Sphericity showed a significance of *P* < 0.001.

### Statistical Analysis

All analyses were carried out using the R (v3.2.5) software. Frequencies and percentages were summarized for the qualitative variables. For quantitative variables, mean and standard deviation (SD) were calculated. Comparisons of demographic and psychological variables between different groups were analyzed using the binary logistic regression for qualitative variables. Odds ratios (OR) and 95% confidence intervals (95% CI) were reported. All *P*-values were two-tailed with a significance level at .05.

## Results

A total of 8,879 questionnaires were included in the analysis after quality audit, yielding an effective rate of 98.2%. Among 8,879 students from universities in 14 cities, 4,834 (54.4%) were female and 7,564 (85.2%) were undergraduate students. The mean and standard deviation of participants' age were 21.27 and 2.39, respectively. A total of 1,941 (21.9%) of the students lived in Hubei Province during the COVID-19 epidemic. The mean and standard deviation of knowledge, attitude, and practice scores were 6.79 (0.99), 4.96 (1.23), and 7.87 (1.52), respectively. Thirty-three (0.4%) participants reported that they or their family members were infected by COVID-19. Moreover, 615 students (6.9%) reported PTSD symptoms, and 465 (5.2%) reported depressive symptoms. The number of students with anxiety symptoms was 896 (10.1%). Additionally, 2,519 students (28.4%) showed problematic Internet use (Table 1 and Supplementary Figure 1).

**Table 1:** Characteristics of participants.

**Characteristic**	**Participants No**.	**%**
**Age (years), mean (SD)**	21.27	2.39
**Sex**		
Male	4,045	45.6
Female	4,834	54.4
**Medical students**		
Yes	5,426	61.1
No	3,453	38.9
**Location during the COVID-19**		
Cities in Hubei province	1,941	21.9
Cities outside Hubei province	6,910	77.8
Abroad	28	0.3
**District**		
Urban	5,084	57.3
Rural	3,795	42.7
**Type of students**		
Undergraduates	7,564	85.2
Master's degree candidates	1,133	12.8
Doctoral candidates	182	2.0
**Anyone around you infected with COVID-19**		
**Yourself or your family**		
Yes	33	0.4
No	8,846	99.6
**Relatives but not live together**		
Yes	59	0.7
No	8,820	99.3
**People in the same community or village**		
Yes	639	7.2
No	8,240	92.8
**Knowledge scores, mean (SD), max** **=** **8**	6.79	0.99
**Attitude scores, mean (SD), max** **=** **8**	4.96	1.23
**Practice scores, mean (SD), max** **=** **9**	7.87	1.52
**Problematic Internet use**		
Yes	2,519	28.4
No	6,360	71.6
**PCL-5**		
PTSD	615	6.9
No PTSD	8,264	93.1
**CES-D9**		
DS	465	5.2
No DS	8,414	94.8
**GAD-7**		
AS	896	10.1
No AS	7,983	89.9
**Total**	8,879	100.0

*COVID-19, coronavirus disease 2019; SD, standard deviation; PTSD, the posttraumatic stress disorder; DS, depressive symptoms; AS, anxiety symptoms; PCL-5, the PTSD Checklist for DSM-5; CES-D9, the Center for Epidemiologic Studies Depression 9-item scale; GAD-7, the generalized anxiety disorder 7-item scale*.

Students who showed problematic Internet use, compared with those who did not show problematic Internet use, had an increased risk of PTSD, depressive, and anxiety symptoms (odds ratio 2.662 [95% CI, 2.239–3.165], odds ratio 4.582 [95% CI, 3.753–5.611], odds ratio 3.251 [95% CI, 2.814–3.757], respectively). When considering the four clusters of PCL-5, the independent samples *t*-tests also indicated that students with problematic Internet use showed more PTSD symptoms than those without problematic Internet use on B, C, D, and E clusters in PCL-5 (*P* < 0.001) (Tables 2–4 and Supplementary Figure 2). Moreover, we found that male students, students who were infected or whose families were infected with COVID-19, those who had lower attitude scores and practice scores, non-medical students, and master's degree candidates had an increased risk of PTSD, depressive, or anxiety symptoms (Tables 2–4).

**Table 2:** Characteristics of participants according to the posttraumatic stress disorder symptoms.

**Characteristic**	**PTSD No. (%)**^a^	**OR (95% CI)**	***P*-value**
**Age (years), mean (SD)**	21.16 (2.29)	1.026 (0.969, 1.084)	0.36
**Sex**			
Male	372 (9.2)	1 (Reference)	NA
Female	243 (5.0)	0.511 (0.425, 0.614)	<0.001
**Medical students**			
Yes	359 (6.6)	1 (Reference)	NA
No	256 (7.4)	0.889 (0.721, 1.095)	0.27
**Location during the COVID-19**			
Cities in Hubei province	131 (6.7)	1 (Reference)	NA
Cities outside Hubei province	482 (7.0)	1.177 (0.935, 1.493)	0.17
Abroad	2 (7.1)	0.578 (0.032, 2.860)	0.60
**District**			
Urban	329 (6.5)	1 (Reference)	NA
Rural	286 (7.5)	1.064 (0.892, 1.269)	0.49
**Type of students**			
Undergraduates	553 (7.3)	1 (Reference)	NA
Master's degree candidates	51 (4.5)	0.518 (0.352, 0.752)	0.001
Doctoral candidates	11 (6.0)	0.757 (0.347, 1.519)	0.46
**Anyone around you infected with COVID-19**			
**Yourself or your family**			
Yes	7 (21.2)	3.686 (1.400, 8.623)	0.004
No	608 (6.9)	1 (Reference)	NA
**Relatives but not live together**			
Yes	6 (10.2)	1.122 (0.365, 2.781)	0.82
No	609 (6.9)	1 (Reference)	NA
**People in the same community or village**			
Yes	44 (6.9)	1.147 (0.803, 1.607)	0.44
No	571 (6.9)	1 (Reference)	NA
**Knowledge scores, mean (SD)**	6.70 (1.22)	0.930 (0.856, 1.012)	0.09
**Attitude scores, mean (SD)**	4.55 (1.46)	0.745 (0.696, 0.797)	<0.001
**Practice scores, mean (SD)**	7.55 (1.88)	0.939 (0.890, 0.992)	0.02
**Problematic Internet use**			
Yes	305 (12.1)	2.662 (2.239, 3.165)	<0.001
No	310 (4.9)	1 (Reference)	NA

*COVID-19, coronavirus disease 2019; NA, not applicable; SD, standard deviation; PTSD, the posttraumatic stress disorder*.

a *The number and percentage of the students who reported PTSD symptoms*.

**Table 3:** Characteristics of participants according to the depressive symptoms.

**Characteristic**	**DS No.(%)**^**a**^	**OR (95% CI)**	***P*-value**
**Age (years), mean (SD)**	21.10 (2.27)	1.016 (0.950, 1.083)	0.63
**Sex**			
Male	220 (5.4)	1 (Reference)	NA
Female	245 (5.1)	1.019 (0.828, 1.255)	0.86
**Medical students**			
Yes	227 (4.2)	1 (Reference)	NA
No	238 (6.9)	1.504 (1.189, 1.903)	0.001
**Location during the COVID-19**			
Cities in Hubei province	107 (5.5)	1 (Reference)	NA
Citites outside Hubei province	358 (5.2)	1.325 (1.022, 1.733)	0.04
Abroad	0 (0)	0.000 (0.000, 2.851)	0.96
**District**			
Urban	285 (5.6)	1 (Reference)	NA
Rural	180 (4.7)	0.861 (0.702, 1.054)	0.15
**Type of students**			
Undergraduates	395 (5.2)	1 (Reference)	NA
Master's degree candidates	63 (5.6)	0.994 (0.677, 1.455)	0.97
Doctoral candidates	7 (3.8)	0.776 (0.297, 1.765)	0.57
**Anyone around you affected with COVID-19**			
**Yourself or your family**			
Yes	3 (9.1)	0.977 (0.153, 3.448)	0.98
No	462 (5.2)	1 (Reference)	NA
**Relatives but not live together**			
Yes	4 (6.8)	0.962 (0.274, 2.588)	0.95
No	461 (5.2)	1 (Reference)	NA
**People in the same community or village**			
Yes	54 (8.5)	1.612 (1.134, 2.258)	0.01
No	411 (5.0)	1 (Reference)	NA
**Knowledge scores, mean (SD)**	6.91 (1.06)	1.142 (1.033, 1.266)	0.01
**Attitude scores, mean (SD)**	4.48 (1.60)	0.765 (0.709, 0.825)	<0.001
**Practice scores, mean (SD)**	7.50 (1.82)	0.883 (0.833, 0.938)	<0.001
**Problematic Internet use**			
Yes	299 (11.9)	4.582 (3.753, 5.611)	<0.001
No	166 (2.6)	1 (Reference)	NA

*COVID-19, coronavirus disease 2019; NA, not applicable; SD, standard deviation; DS, depressive symptoms*.

a *The number and percentage of the students who reported depressive symptoms*.

**Table 4:** Characteristics of participants according to the anxiety symptoms.

**Characteristic**	**AS No. (%)**^**a**^	**OR (95% CI)**	***P*-value**
**Age (years), mean (SD)**	21.25 (2.23)	1.060 (1.012, 1.110)	0.01
**Sex**			
Male	447 (11.1)	1 (Reference)	NA
Female	449 (9.3)	0.847 (0.727, 0.987)	0.03
**Medical students**			
Yes	493 (9.1)	1 (Reference)	NA
No	403 (11.7)	1.192 (1.000, 1.419)	0.05
**Location during the COVID-19**			
Cities in Hubei province	212 (10.9)	1 (Reference)	NA
Citites outside Hubei province	683 (9.9)	1.015 (0.842, 1.228)	0.88
Abroad	1 (3.6)	0.335 (0.019, 1.627)	0.29
**District**			
Urban	508 (10.0)	1 (Reference)	NA
Rural	388 (10.2)	1.010 (0.871, 1.170)	0.90
**Type of students**			
Undergraduates	772 (10.2)	1 (Reference)	NA
Master's degree candidates	108 (9.5)	0.716 (0.537, 0.952)	0.02
Doctoral candidates	16 (8.8)	0.633 (0.329, 1.153)	0.15
**Anyone around you affected with COVID-19**			
**Yourself or your family**			
Yes	5 (15.2)	1.282 (0.417, 3.236)	0.63
No	891 (10.1)	1 (Reference)	NA
Relatives but not live together			
Yes	10 (16.9)	1.556 (0.702, 3.152)	0.25
No	886 (10.0)	1 (Reference)	NA
**People in the same community or village**			
Yes	71 (11.1)	0.948 (0.705, 1.259)	0.72
No	825 (10.0)	1 (Reference)	NA
**Knowledge scores, mean (SD)**	6.83 (1.08)	1.059 (0.984, 1.140)	0.13
**Attitude scores, mean (SD)**	4.57 (1.48)	0.773 (0.730, 0.818)	<0.001
**Practice scores, mean (SD)**	7.56 (1.82)	0.896 (0.857, 0.938)	<0.001
**Problematic Internet use**			
Yes	489 (19.4)	3.251 (2.814, 3.757)	<0.001
No	407 (6.4)	1 (Reference)	NA

*COVID-19, coronavirus disease 2019; NA, not applicable; SD, standard deviation; AS, anxiety symptoms*.

a *The number and percentage of the sudents who reported anxiety symptoms*.

## Discussion

In the present study, we reported the prevalence of PTSD, depressive, and anxiety symptoms to be 6.9, 5.2, and 10.1%, respectively and problematic Internet use was significantly associated with depressive, anxiety and PTSD symptoms, as well as the B, C, D, and E clusters of PCL-5. Lower attitude scores and practice scores also contributed to the risk of depressive, anxiety, and PTSD symptoms.

The detection rate of PTSD symptoms was similar to Liu's study (7%) (8) and Chew's study (7.4%) (50) and was lower compared to other recently published studies (10.8–27.39%) (9, 12, 15, 51). However, our result was higher than the study focusing on Chinese university students (2.7%) (18). Tang et al. investigated the mental health problems of students mainly from universities in Chengdu and Chongqing ~1 month after the COVID-19 outbreak in China, which was earlier than our study. The higher non-response rate and psychological resistance in the acute stage of trauma may lead to the underestimation of PTSD (52). Based on previous studies about the SARS epidemic, the longer isolation durations were significantly associated with higher possibility of psychological distress such as PTSD and depression (5, 53). Moreover, 0.4% of students in our study reported that they or their family members were infected with COVID-19, which was 100 times higher than in Tang's research (1/2,485). We also found that family members infected with COVID-19 were associated with increased risk of PTSD symptoms. The severer exposure to the stress event — COVID-1 9— may partly explain the higher PTSD prevalence in our study (54). In consequence, PTSD symptoms should be paid more attention, even if COVID-19 is well-controlled now and schools reopen step by step. Students who experience a more serious traumatic event need additional attention.

The detection rate of depressive and anxiety symptoms was lower than in recently published studies (55) and higher than in Tan's study (3.7 and 3.8%, respectively) (12). For Chinese university students, Chang et al. (17) found that 4.18 and 3.41% of students experienced moderate to severe depressive and anxiety symptoms, respectively, which were lower than in our study. The detection rate of anxiety symptoms in our study was higher than a study focusing on Chinese medical college students (3.6%) (56). The students in Chang's study were all from universities in Guangdong Province, China, while more than half of the participants in our study were recruited from Hubei Province, which was the epidemic center with more than 50,000 patients. Besides, the proportion of medical students in Chang's study was higher compared to our study. Medical students, usually having a better understanding of this disease, may stay rational when faced with the sheer number of sensationalized news headlines and erroneous news reports, which could contribute, in turn, to a reduction in anxiety (57). Furthermore, medical students tended to show a higher level of resilience, which was positively correlated with adaptive coping strategies when facing problems and had been shown to prevent the development of PTSD, anxiety, and depression (58). Another study showed that anxiety levels of medical students significantly decreased after switching to online learning, in contrast with their non-medical peers (59). This may be due to the lightening of an overloaded academic curriculum. In our study, non-medical students had significantly increased risk of depressive and anxiety symptoms. The depressive and anxiety symptoms of students, especially non-medical students, deserves attention.

The result indicated that there was a strong association between problematic Internet use and PTSD, depressive, and anxiety symptoms. Data from the China Internet Network Information Center (CNNIC), as of March 15, 2020, showed that 904 million people had gone online, of which 26.9% were students (60). Students obtained a lot of epidemic-related information from the Internet, which tended to be full of rumors and uncertainty in initial disease outbreak periods. Such experience may amplify students' psychological distress and contribute to the occurrence of psychological problems. Beyond that, addictive behaviors often function as regulation strategies of the psychological problem of maladjustment, such as depression and anxiety (24). On the other hand, considering problematic Internet use behaviors like behavioral addiction, it shares similar basic mechanisms with substance dependence (61). Substance abuse (e.g., drugs and alcohol) may disturb a person's ability to cope with trauma effectively, leading to long-term symptoms of PTSD (62). In turn, subjects exposed to trauma may depend on substance abuse in attempts to manage symptoms, hyperarousal, flashbacks, or painful memories, to name a few (63). Besides, non-adaptive/negative thinking styles, which usually correlate with mental health problems such as depression and suicide ideation, are found in subjects who show addictive problematic Internet use (64, 65). Samples with problematic Internet use reported a greater risk of avoidant tendencies (66), which may be a feature of PTSD (67), and positive coping and adaptive strategies could moderate the development of PTSD symptoms in trauma-exposed subjects (68). Considering the association between problematic Internet use and psychological problems, an evaluation for problematic Internet use may be included in the assessment of individuals suspected of PTSD, depression, and anxiety to enhance the screening capability.

Our study found that females had a lower risk of PTSD and anxiety symptoms, in contrast with previous studies (8–10). However, Liang et al. (15) and Jin et al. (69) found a similar result. Males may be more likely than women to handle adverse experiences through aggression and addictive behaviors (70–72), which was also indicated in our study (The problematic Internet use of males vs. females: 29.0% [1,172/4,045] vs. 27.9% [1,347/4,834]). A study utilizing functional magnetic resonance imaging indicated that men may experience increased anxiety in response to stress because of hypoactivation, or even suppression, of certain brain regions (i.e., left temporal gyrus, cerebellum) (73). Other researchers stated that femininity (affection, compassion, and sensitivity to others' needs) appeared to be protective against depression symptoms in college-educated people (74). On the other hand, the proportion of female medical students was 74.9% (3,623/4,834), compared to only 44.6% of male medical students (1,803/4,045) in our study. Compared with undergraduate students, master's degree candidates showed lower risk of PTSD and anxiety symptoms, which was consistent with a previous study (75). An explanation of this result is that older students with a higher educational level may have more experience and better coping strategies in dealing with threats (76). Meanwhile, young adult students, who were in the process of achieving important development milestones, faced academically-related stressors and were particularly vulnerable to psychological distress (75). We also found that higher scores of attitude and practice were protective factors for PTSD, depressive, and anxiety symptoms, which was similar to other studies (77). The KAP toward infectious diseases may be associated with mental health by affecting the level of panic and stress emotions in the population. It is recommended to make the most of the online health education services in China. This may facilitate the improvement of KAP levels of COVID-19 and alleviate the adverse psychological impacts and psychiatric symptoms of students.

There are some limitations in the present study. Information on the psychological status of participants before the COVID-19 outbreak is lacking. Moreover, the cross-sectional design could not evaluate whether psychological problems will be long-lasting after the COVID-19 outbreak. Follow-up studies with these participants will help us understand how long the symptoms will last and guide future intervention aimed at university students. In addition, some characteristics in problematic Internet use may be essential in the development of psychological problems, and the association needs to be further studied in samples exposed to COVID-19. The YDQ might bias the assessment of problematic Internet use, as the tool is somewhat outdated. Although the YDQ is still used in many studies (78–81), an updated model might be more suitable for the investigation of the Internet use (i.e., the Interaction of Person–Affect–Cognition–Execution model) (82). Finally, the snowball sampling method may result in a biased sample. Selection bias may also lead to the findings that psychological problems of university students are related to gender, grade and other factors, which need to be verified in a larger sample in the future.

In conclusion, our study, with a large sample of Chinese university students, identified a 6.9, 5.2, and 10.1% prevalence of PTSD, depressive, and anxiety symptoms, respectively, ~3 months after the COVID-19 outbreak. We also found a strong association between problematic Internet use and PTSD, depressive, and anxiety symptoms. Considering that psychological problems among university students were common before the epidemic, although, COVID-19 will aggravate the psychological problems of students, the existing academic pressure and career pressure should not be ignored. Our results could complement the current state of mental health among university students during the COVID-19 period. Additionally, how mental health consequences can be mitigated under pandemic conditions is a priority that the researchers highlight (83). When it comes to mental health issues, problematic Internet use also needs attention. In addition, our findings could also assist in identifying college students with an elevated risk of psychological problems and universities could consider planning for long-term psychological services for these students. Interventions such as improving the KAP toward COVID-19 would also decrease the risk of psychological problems.

## Data Availability Statement

The datasets presented in this article are not readily available because participants of this study did not agree for their data to be shared publicly; therefore, supporting data are not available. Requests to access the datasets should be directed to Ranran Song, songranran@hust.edu.cn.

## Ethics Statement

The studies involving human participants were reviewed and approved by Ethics Committee of Tongji Medical College, Huazhong University of Science and Technology. The statement ‘I agree to participate in the survey voluntarily' was presented in the survey link. The students proceeded to the survey after they had consented.

## Author Contributions

XX, BZ, and RS conceived the study. XX, KZ, JZ, and RS critically appraised the data. XX, KZ, and QX prepared the initial manuscript. BZ and RS reviewed and edited the manuscript. All authors have collected data for the study, critically reviewed, and approved the final manuscript.

## Conflict of Interest

The authors declare that the research was conducted in the absence of any commercial or financial relationships that could be construed as a potential conflict of interest.
