# Supplementary material for: Problematic Internet Use Was Associated With Psychological Problems Among University Students During COVID-19 Outbreak in China
Source: Front Public Health. 2021 Jun 15;9:675380. doi: 10.3389/fpubh.2021.675380 (PMC8239128; doi:10.3389/fpubh.2021.675380)
Supplement: Supplementary file 1 [file Data_Sheet_1.docx]

**Methods**

This study was approved by the Ethics Committee of Tongji Medical College, Huazhong University of Science and Technology (protocol number: S104). The informed consent was obtained from the participants before the investigation. An ID was randomly given to each participant. There were no disclose information that might identify a particular person. All procedures performed in studies involving human participants were following the 1964 Helsinki declaration and its later amendments.

A snowball sampling method was used to invite undergraduate and graduate students to participant the online survey between April 20 and April 26, 2020, approximately three months after the COVID-19 outbreak in China. Survey link was distributed in the class group through the head teacher. Participants voluntarily completed this open survey through an online crowdsourcing platform called Wenjuanxing in mainland China, which provides functions equivalent to Amazon Mechanical Turk. The survey link was sent to the student’s cellphone and statement “I agree to participate in the survey voluntarily” was presented to the participant before the survey. The students proceeded to the survey after they had consented. The usability and technical functionality of the electronic questionnaire had been tested before distributing the questionnaire. No incentives were offered to the participants. The data was stored on a dedicated computer with a password.

The questionnaire was divided into 5 pages, with about 7 questionnaire items per page. Most of questionnaire items were compulsory. Only after these items were completed can the questionnaire be submitted. During the questionnaire process, respondents were able to review and change their answers. Only one questionnaire can be submitted from the same IP address. When analyzing the data, only completed questionnaires were involved and questionnaires with less than 5 minutes filling time were excluded.

**Code of analyses in this study.**

a = read.csv('dataset for R.csv')

a$sex = factor(a$sex, levels = c('1', '2'))

a$medical = factor(a$medical, levels = c('1','2'))

a$location = factor(a$location, levels = c('1','2','3'))

a$district = factor(a$district, levels = c('1','2'))

a$studenttype= factor(a$studenttype, levels = c('1','2','3'))

a$yourselfaffected = factor(a$yourselfaffected, levels = c('0','1'))

a$relativeaffected = factor(a$relativeaffected, levels = c('0','1'))

a$communityaffected = factor(a$communityaffected, levels = c('0','1'))

a$internetaddiction = factor(a$internetaddiction, levels = c('0','1'))

a$depressiongroup = factor(a$depressiongroup, levels = c('0','1'))

a$anxietygroup = factor(a$anxietygroup, levels = c('0','1'))

### logistic regression ###

glm1<-glm(anxietygroup~sex+age+medical+location+district+studenttype+yourselfaffected+relativeaffected+communityaffected+Kscore+Ascore+Pscore+internetaddiction, family = binomial(link=logit), data=a)

summary(glm1)

CI <- data.frame(exp(confint(glm1)))

exp(coef(glm1))

results_logistic <- data.frame(summary(glm1)[["coefficients"]])

results_logistic$beta <- exp(results_logistic$Estimate)

results_logistic <- cbind(results_logistic, CI)

glm2<-glm(depressiongroup~sex+age+medical+location+district+studenttype+yourselfaffected+relativeaffected+communityaffected+Kscore+Ascore+Pscore+internetaddiction, family = binomial(link=logit), data=a)

summary(glm2)

CI <- data.frame(exp(confint(glm2)))

exp(coef(glm2))

results_logistic <- data.frame(summary(glm2)[["coefficients"]])

results_logistic$beta <- exp(results_logistic$Estimate)

results_logistic <- cbind(results_logistic, CI)

glm3<-glm(ptsdgroup~sex+age+medical+location+district+studenttype+yourselfaffected+relativeaffected+communityaffected+Kscore+Ascore+Pscore+internetaddiction,family = binomial(link=logit), data=a)

summary(glm3)

CI <- data.frame(exp(confint(glm3)))

exp(coef(glm3))

results_logistic <- data.frame(summary(glm3)[["coefficients"]])

results_logistic$beta <- exp(results_logistic$Estimate)

results_logistic <- cbind(results_logistic, CI)

Supplementary Table 1 Scoring method of questions about knowledge, attitudes and practices

| **Questions** | **Contents** | **Scoring method** | **Score range** |
| --- | --- | --- | --- |
| Knowledge K1 | Which of the following is the possible route of SARS-CoV-2 transmission? | add 1 point for "respiratory droplets", "contact transmission", "fecal-oral transmission", and "aerosol transmission". Add 0 point for "blood transmission" and "I don't know". | 0-4 |
| Knowledge K2 | Which of the following prevention is ture? | add 1 point for each checked option, including "Wearing a mask when go out", "Washing hands frequently", "Ventilating the house frequently", "Avoiding crowded places". | 0-4 |
| Attitude A1 | Do you worried about being infected with COVID-19? | "Quite worried' 0 point, 'Moderately worried" 1 point, "Slightly worried" 2 point, "Rarely worried" 3 point, "Not at all worried" 4 point. | 0-4 |
| Attitude A2 | What is your attitudes towards the COVID-19 epidemic? | "Quite pessimistic" 0 point, "Moderately pessimistic" 1 point, "Indifferent" 2 point, "Moderately optimistic" 3 point, "Quiet optimistic" 4 point. | 0-4 |
| Practice P1 | Which of the following situation you will wash your hands? | add 1 point for each checked option, including ''After using the bathroom", "After getting home", "After coughing or sneezing", "Before having a meal", "After touching the object outside home". "I would not wash my hands on purpose", 0 point. | 0-5 |
| Practice P2 | What have you done during the outbreak? | add 1 point for each checked option, including ''Sharing the information of COVID-19 with others", "Reminding my family members to wear masks", "Ventilating the house frequently", "Washing hands frequently". "None of the above", 0 point. | 0-4 |


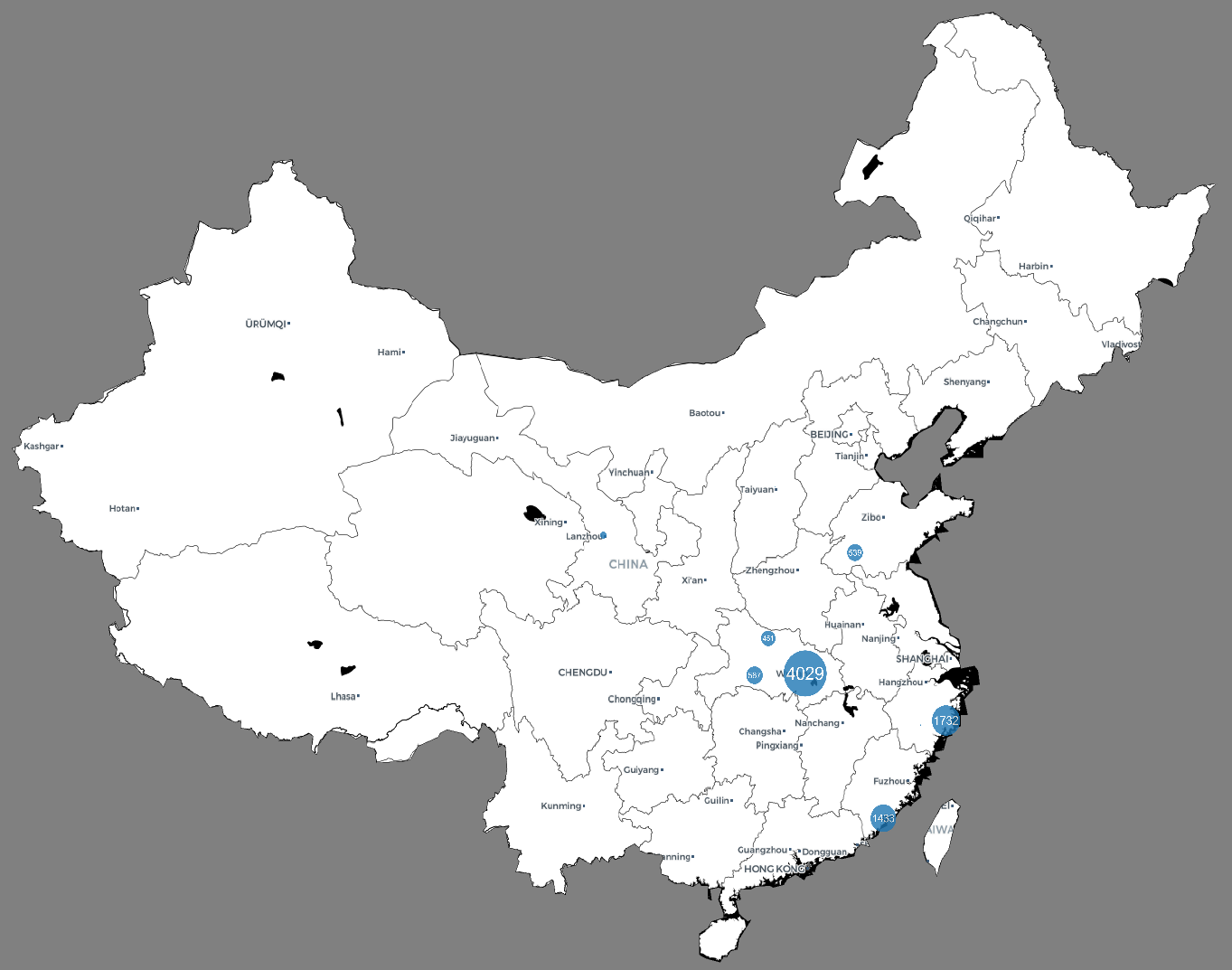


**Supplementary Figure 1** **The distribution of the universities of participants.** The numbers in the blue circle represent the number of students in the city. The participants were from universities in 14 cities across China.


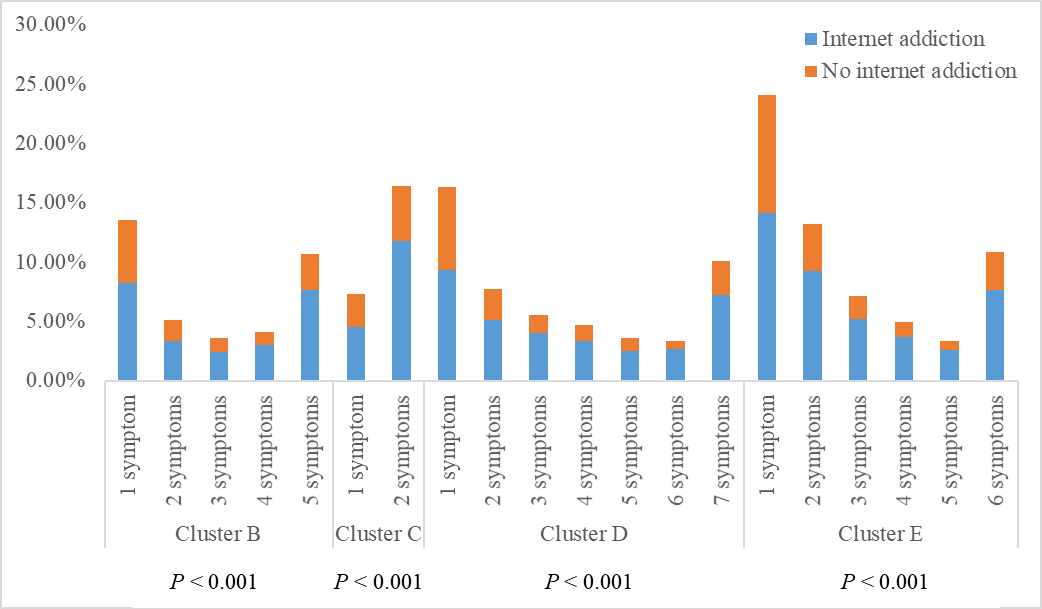
**Supplementary Figure 2 Prevalence differences of the posttraumatic stress disorder (PTSD) symptoms on four cluster between students with problematic Internet use and without problematic Internet use.** 1 symptom represented only one item in the cluster that participants rated 2 or higher and 2 symptoms represented 2 items in the cluster that participants rated 2 or higher. Independent samples t-tests was used to compare the differences in the prevalence of PTSD symptoms on four cluster students with problematic Internet use and without problematic Internet use. In the four clusters, all *P* values were less than 0.001, indicating that students with problematic Internet use showed more PTSD symptoms than those without problematic Internet use on B, C, D and E clusters.
